# Population Genomics of the Neotropical Brown Stink Bug, *Euschistus heros*: The Most Important Emerging Insect Pest to Soybean in Brazil

**DOI:** 10.3389/fgene.2019.01035

**Published:** 2019-10-31

**Authors:** Maria I. Zucchi, Erick M. G. Cordeiro, Xing Wu, Letícia Marise Lamana, Patrick J. Brown, Shilpa Manjunatha, João Paulo Gomes Viana, Celso Omoto, José B. Pinheiro, Steven J. Clough

**Affiliations:** ^1^Institute of Biology, University of Campinas, Campinas, Brazil; ^2^Agência Paulista de Tecnologia dos Agronegócios, Pólo Regional Centro-Sul, Piracicaba, Brazil; ^3^Department of Entomology, University of São Paulo–ESALQ/USP, Piracicaba, Brazil; ^4^Department of Crop Sciences, University of Illinois at Urbana-Champaign, Urbana, IL, United States; ^5^Department of Fitotecnia e Fitossanidade, Universidade Estadual de Ponta Grossa, Ponta Grossa, Brazil; ^6^Department of Genetics, University of São Paulo–ESALQ/USP, Piracicaba, Brazil; ^7^US Department of Agriculture, Agricultural Research Services, Soybean/Maize Germplasm, Pathology, and Genetics Research Unit, Urbana, IL, United States

**Keywords:** soybean pest, genotyping by sequencing, population genomics, single-nucleotide polymorphism marker, *Euschistus heros*, Neotropical brown stink bug

## Abstract

Recent changes in soybean management like the adoption of transgenic crops and no-till farming, in addition to the expansion of cultivated areas into new virgin frontiers, are some of the hypotheses that can explain the rise of secondary pests, such as the Neotropical brown stink bug, *Euschistus heros*, in Brazil. To better access the risk of increased pests like *E. heros* and to determine probabilities for insecticide resistance spreading, it is necessary first to access the levels of the genetic diversity, how the genetic diversity is distributed, and how natural selection is acting upon the natural variation. Using the genotyping by sequencing (GBS) technique, we generated ~60,000 single-nucleotide polymorphisms (SNPs) distributed across the *E. heros* genome to answer some of those questions. The SNP data was used to investigate the pattern of genetic structure, hybridization and natural selection of this emerging pest. We found that *E. heros* populations presented similar levels of genetic diversity with slightly higher values at several central locations in Brazil. Our results also showed strong genetic structure separating northern and southern Brazilian regions (F_ST_ = 0.22; p-value = 0.000) with a very distinct hybrid zone at the central region. The analyses also suggest the possibility that GABA channels and odorant receptors might play a role in the process of natural selection. At least one marker was associated with soybean and beans crops, but no association between allele frequency and cotton was found. We discuss the implications of these findings in the management of emerging pests in agriculture, particularly in the context of large areas of monoculture such as soybean and cotton.

## Introduction

Habitats and landscapes featuring extensively cultivated areas foist new challenges upon ecologists and populations geneticists every time a new technology emerges and is incorporated into the set of pest management tactics. The implementation of such technologies—including no-till farming, novel pesticides, and transgenic crops—modify the agricultural conditions for resident insect populations setting the stage for the potential rise of new pests problems (Estruch et al., 1997; Corrêa et al., 2019). Albeit not easy to accomplish, we have much to gain from the early detection of changes in the pest ecological dynamics (e.g., changes in species dominance and geographic range expansion) and in the evolutionary trajectories of important adaptations (e.g., dormancy, spectrum of polyphagia, and insecticide resistance) (Simberloff and Stiling, 1996; Anderson et al., 2018). These problems can become yet more critical and tend to occur first in places where agriculture is practiced intensively and in large scale due to a fierce natural selection pressure imposed by the environment. Brazilian agriculture is both technologically intensive (i.e., three crop cycles per year in some places) and scale-intensive (i.e. performed over very large acreage), which lead to great challenges when it comes to pest control. Although technological innovations have led to steadily increasing yields for crops like soybean, farmers still experience insect control failure more often than would be desirable (Sosa-Gómez and Silva, 2010; Blackman et al., 2015; Tuelher et al., 2018).

Throughout the world, the development and adoption of transgenic *Bt* crops represent an important landmark in insect control, but the use of *Bt* has also been linked to an increase in the densities of sap-sucking insects, in particular, hemipterans (Wang et al., 2008; Saluso et al., 2011; Zhao et al., 2011; Catarino et al., 2015; Viktorov, 2017). Transgenic *Bt* crops have been very effective against lepidopteran insects, allowing growers to apply less insecticide. However, this decrease in insecticide use, coupled with the negligible effect of *Bt* on hemipterans, has been attributed as one of the main causes of hemipterans outbreaks in soybean fields. In Brazil, this increase in hemipterans population densities has been more apparent in the region of Cerrado (the Brazilian savanna), located at the central portion of the country and where soybean has been produced intensively and in large-scale in the last 40 years (Spehar, 1995). Not coincidently, the Brazilian Cerrado is also the place where the most intensive cotton-farming can be found, often side-by-side with soybean or rotated on the same land. Considering all the aspects mentioned, we can formulate the following questions. Is there an evolutionary change in the Hemiptera pest populations in Brazil? If so, what are the underlying details of this evolutionary process? (Tabashnik, 1983; Holt and Gaines, 1992).

The stink bug complex (Hemiptera: Pentatomidae) is composed of approximately 4,123 species distributed around the world from which we can highlight *Piezodorus guilidinii* (W.), *Nezara viridula* (L.), and *Euschistus heros* (F.) for their major importance in agricultural ecosystems (Smaniotto and Panizzi, 2015). *Euschistus heros*, in particular, has been drawing great attention recently in Brazil. Considered endemic to the neotropical regions and with restricted distribution, *E. heros* is now reportedly expanding its native geographic range and distribution and is now frequently associated with losses in soybean crops grown in South America (Saluso et al., 2011; Panizzi, 2015; Soares et al., 2018). Not only that, but *E. heros* feeding and dormancy behaviors have been reportedly changing, which instigates speculations about the ongoing selection of novel traits in the crop fields. This species is capable of partial dormancy without feeding (extend up to six months) that can be considered valuable adaptation for feeding on annual crops such as soybean (Panizzi and Vivan, 1997). Additionally, there are also reports of *E. heros* activity moving over plant biomass that is left on the top of the soil surface during intercropping periods, and a growing number of insect captured in cotton suggest a recent change in the pest dynamics (Sosa-Gómez and Silva, 2010; de Freitas Bueno et al., 2015; Soria et al., 2017). Underlying the recent behavioral changes, no-till farming (by creating suitable dormancy sites), rotations providing suitable hosts, and the admixture of the two genetically distant lineages (i.e., population hybridization) might all be contributing to the problem.

Two divergent *E. heros* lineages were uncovered in a recent phylogeographic study using mitochondrial markers that showed that the pattern of the spatial distribution of the two groups is still greatly preserved to this day (Soares et al., 2018). The northern lineage showed higher haplotype diversity, whereas the southern lineage had lower haplotype diversity. High levels of genetic diversity found using both the mtDNA (Soares et al., 2018) and nDNA (Husch et al., 2018) in secondary contact putative areas compared to the pure-bred areas, suggests hybridization between the two lineages. However, currently, the potential for population admixture between the two lineages have not been accessed. Historic events likely caused the allopatric separation of the two lineages in the distant past leaving marks in the present population, but recurrent events, such as the isolation by distance, has also been suggested to be a significant force explaining the current pattern of diversity distribution (Husch et al., 2018). A comprehensive approach is necessary to investigate the importance of recurrent gene flow on a wider scale and the impacts of the admixture of the two lineages.

*Euschistus heros* has gained more attention in the last decade because of its frequent outbreaks in soybean fields and increasing presence in cotton (Sosa-Gómez and Silva, 2010; de Freitas Bueno et al., 2015; Soria et al., 2017). The need to understand which factors have caused the striking changes in population dynamics have sparked recent studies with this insect group (Husch et al., 2018; Soares et al., 2018). However, higher resolution markers can help us understand which adaptive processes are taking place in the soybean and cotton crop fields (Baird et al., 2008). Thus, we used genotyping-by-sequencing (GBS) to generate thousands of genomic markers for investigation of the population genomics of this species. The main objective of this study was to characterize the levels of diversity in different regions in Brazil, and how diversity is distributed across different soybean and cotton-producing regions. We also investigated possible candidate genes for selection and their functional ontology. We present the first study that compares the genomic variation of *E. heros* at the genomic level.

## Materials and Methods

### Study Sites

**Figure 1:**
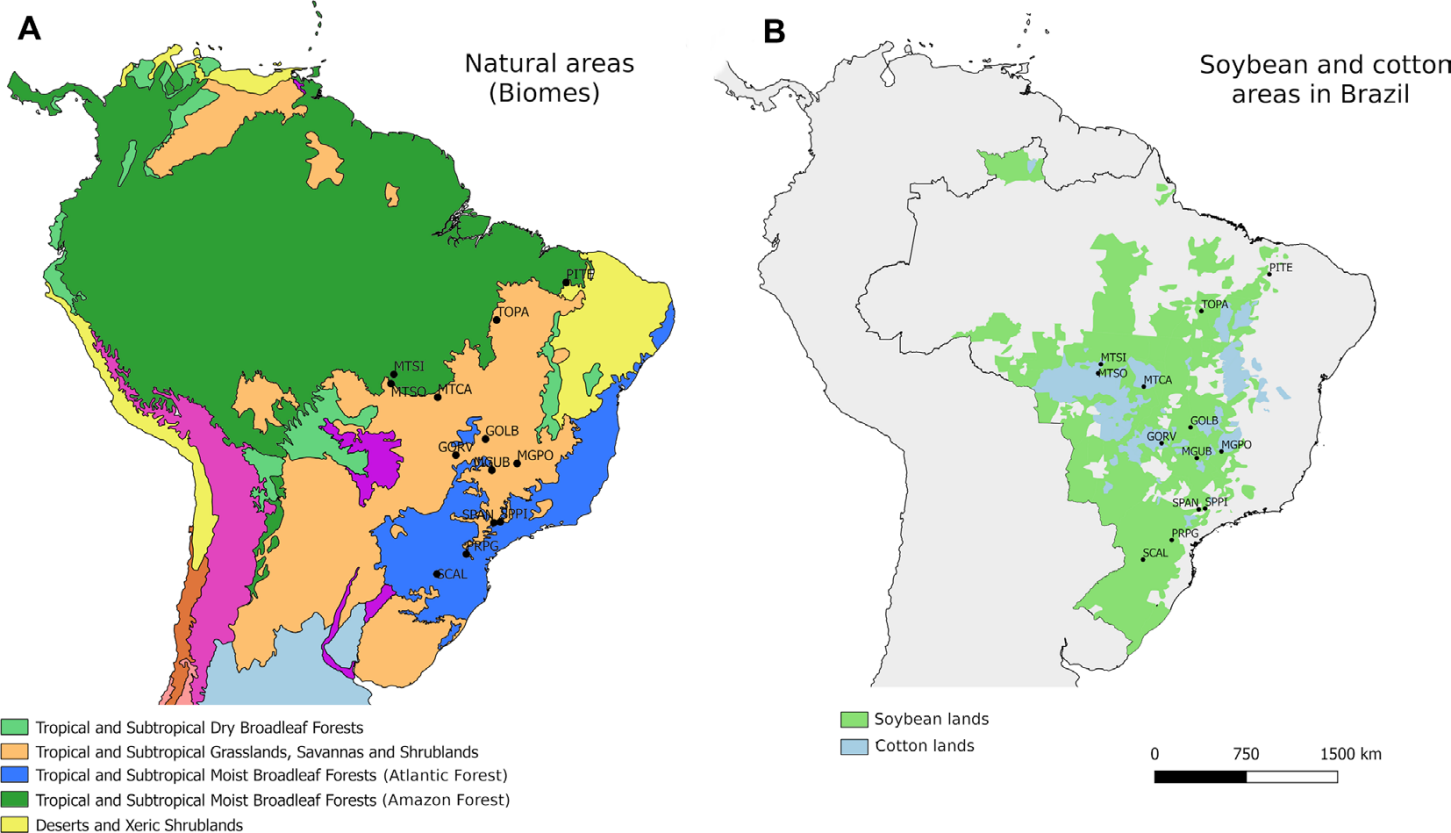
*Euschistus heros* sampling locations in Brazil according to **(A)** ecoregions and **(B)** soybean (green) or soybean + cotton (blue) regions. Map displays the ranges of geographic distribution all soybean and cotton harvested in 2016 (source: IBGE 2016).

For the genomic characterization, a total of 192 individuals of *E. heros* were collected from 13 locations covering the main soybean and cotton-producing areas and the main habitat type (i.e., biomes) in Brazil (**Table 1**; **Figure 1**). Samples were collected between the period 02/2016 and 04/2016, which correspond to the end of the soybean reproductive stage when bugs are more frequently observed. Upon collection, sampled were immersed in 98% EtOH and stored at −80°C until DNA extraction.

**Table 1:** Sampling location information on ecoregion, location, coordinates, and number of individuals successfully genotyped (N_GEN_).

Ecoregions	Municipalities	Code	Latitude	Longitude	N_GEN_
Atlantic Forest	Abelardo Luz, SC	SCAL	26°33'53’’S	52°19’42’’W	13
Atlantic Forest	Ponta Grossa, PR	PRPG	25°05’42’’S	50°09’43’’W	20
Atlantic Forest	Anhembi, SP	SPAN	22°47’22’’S	48°07’38’’W	17
Atlantic Forest	Piracicaba, SP	SPPI	22°43’31’’S	47°38’57’’W	20
Cerrado	President Olegario,MG	MGPO	18°25’04’’S	46°25’05’’W	17
Cerrado	Uberlandia, MG	MGUB	18°55’07’’S	48°16’38’’W	14
Cerrado	Rio Verde, GO	GORV	17°47’53’’S	50°55’41’’W	10
Cerrado	Leopoldo Bulhoes, GO	GOLB	16°37’09’’S	48°44’37’’W	16
Cerrado	Canarana, MT	MTCA	13°33’08’’S	52°16’06’’W	10
Cerrado	Sorriso, MT	MTSO	12°32’43’’S	55°42’41’’W	11
Cerrado	Sinop, MT	MTSI	11°51’51’’S	55°30’09’’W	9
Cerrado	Palmeirante, TO	TOPA	07°51’36’’S	47°55’33’’W	15
Cerrado	Teresina, PI	PITE	05°05’21’’S	42°48’07’’W	20

### DNA Isolation and Genomic Library Preparation

Genomic DNA was isolated from the insects’ head using the modified cetyltrimethylammonium bromide (2%) protocol (Doyle, 1987) with the addition of Proteinase K (Sinapse Biotecnologia). DNA quality and quantity were determined by electrophoresis on agarose gels (1% w/v) stained with SYBR Safe DNA Gel Stain (Invitrogen), and by visual comparison with lambda DNA (Invitrogen).

The reduced representation libraries were prepared according to the protocol of GBS using *PstI* and *MseI* restriction enzymes (Poland et al., 2012). Sequencing was performed on an Illumina HiSeq 4000 with single-reads 150 nt in length. The libraries preparation and sequencing were performed in the Roy J. Carver Biotechnology Center at University of Illinois at Urbana–Champaign (Urbana, IL, USA). Illumina generated reads described in this study is available at the NCBI Sequence Read Archive (SRA) under accession number PRJNA559512.

### Genotyping

Samples were demultiplexed using *process-rad-tags* in STACKS v.2 (Catchen et al., 2013). Illumina reads were trimmed to 90 bp, and a maximum of four mismatches (*M* = 4) and a minimum of three reads were allowed for the stack depth (*m* = 3) to form the RADloci (Ilut et al., 2014). Our filtering criteria to run the population module in STACKS was set to limit missing data to maximum 50% within locations and 50% between location (Hodel et al., 2017). Minor allele frequency (MAF) filter was set to a minimum of 5% (Roesti et al., 2012). More stringent filtering strategies were eventually used to reduce the number of markers and the impact of missing data whenever necessary (Hodel et al., 2017). Outputs were stored in variant call format (VCF), genepop, and structure files. Conversion to other formats, when necessary, were made using PGDSPIDER 2.0 (Lischer and Excoffier, 2012).

### Population Genomic Analysis

Genomic diversity was estimated according to the observed heterozygosity (H_O_), expected heterozygosity (H_E_), nucleotide diversity (π), and the inbreeding coefficients (F_IS_). The genetic differentiation was estimated through the overall and pairwise Fixation Index F_ST_ (Weir and Cockerham, 1984). Visualization of the F_ST_ relations was performed in R using *RColorBrewer* package. We also tested for correlation between the genetic (F_ST_) and the geographic distance using the Mantel test with 10,000 permutations using matrix in three different scales (2500, 1500, and 500 km) using the R library *ecodist* (Goslee and Urban, 2007) and *ade4* (Dray and Dufour, 2007).

Admixture and genetic partition were examined using software STRUCTURE v.2.3.4 (Pritchard et al., 2000; Falush et al., 2003) using a model where admixture and allele frequency correlation were allowed. To run STRUCTURE, we use a subset of loci obtained from a stricter filtering strategy (*p* = 90%, *r* = 75%). The more stringent filtering parameters forced us to remove two populations (i.e., PITE and SCAL) from the dataset due to excess of missing data. The program was set to run 1.5 × 10^5^ burn-in interactions followed by 5 × 10^6^ Markov chain Monte Carlo steps. We simulated the number of clusters ranging from 1 to 15 (K = 1 to K = 15) using 10 replications. Most likely value of K was selected by Evanno method implemented in Structure Harvest (Earl and vonHoldt, 2012). Runs were than averaged in CLUMPP 1.1.2 (Jakobsson and Rosenberg, 2007) and visualized using DISTRUCT (Rosenberg, 2004). Additionally, we investigate levels of genetic structure using principal component analysis (PCA) using *ade4* and *adegenet* in R.

### Outlier SNP Candidate Detection

The strategy for identifying loci putatively under selection was based on the detection of loci at extreme F_ST_ values in relation to expected heterozygosity contrasting observed data to simulated expectations implemented in LOSITAN (Beaumont and Nichols, 1996; Antao et al., 2008). This method describes the expected distribution of F_ST_ in relation to H_E_ (expected heterozygosity) and identifies loci that have excessively high or low F_ST_ compared to expectations under neutrality conditions. LOSITAN was run with 100,000 simulations, with infinite alleles mutation model, 0.995 confident interval and false discovery rate (FDR) of 0.05. Additionally, we used a Bayesian approach to identify candidates using differences in allele frequencies between populations using the software Bayescan (Foll and Gaggiotti, 2008; Fischer et al., 2011). Bayescan uses logistic regression models separating locus effect and population effect, allowing estimated a population-specific F_ST_ coefficient. Runs were set to implement 10 pilot runs of 50,000 interactions and additional burn-in of 50,000 interactions. We used 100,000 interactions (sample size of 5,000 and thinning interval of 20). We also adopted an FDR of 0.05 to select outlier SNP markers in Bayescan. The RADtags in which outlier SNPs were identified were used in nucleotide searches with BLASTx against the genomic database of the National Center for Biotechnology Information (NCBI) using Blast2go. For the sequences with significant BLAST hits, functional annotations were taken using the ontology system “Gene ontology”.

### Association Between Loci and Host Plant Composition in the Landscape

We have tested the association between loci frequencies and the predominance of host plants [soybean (*Glycine max*), common bean (*Phaseolus vulgaris*), and cotton (*Gossypium* hirsutum) crops in varies ratios] using a latent factor mixed-effect model (LFMM) implemented in the R package *LEA* (Frichot et al., 2013; Frichot and François, 2015). LFMM algorithm can detect correlations between loci and environmental variables such as crop composition while taking into account the levels of population structure, i.e., latent factors. To determine the best value for the latent factor, first, we used the *‘snmf’* function to estimate the putative number of ancestral populations evaluating different values of K using a cross-entropy criterion to determine the most likely values. K = 2 was detected as the best value for the latent factor, which was confirmed by STRUCTURE analysis. The following step was to perform the association between loci and environmental variable based on soybean, common bean, and cotton prevalence in each area and total production of each crop per unit of area using the *lfmm*.

The information about soybean, beans, and cotton composition referred to the year 2016 was extracted from the online Brazilian statistic database (SIDRA), which is a repository containing statistics from the Brazilian Institute of Geography and Statistics (IBGE). The agriculture census information referred to the three crops was extracted from 5,564 municipalities providing a grid with crop prevalence in the whole territory. The relative yield and presence of each crop per unit of the area were the two standardized metrics used as environmental factors. The crop variables were first scaled (i.e., z-scored) and then compressed onto a lower-dimensional feature subspace using PCA analysis. Considering only the subset of sampled areas, cotton occurrence in the agriculture landscape varied from 0 up to 8.42% (MGPO) in contrast to soybean that varied from 1% (SPAN) to 76.2% (TOPA). Common bean crops were also present in lower proportions in sampled areas varying from ~0.25% (SPPI) to 9% (PITE). In summary, PC1 (39.3%) captured the major differences regarding areas and yield in soybean, PC2 (25.2%) captured differences associated with cotton and PC3 (13.8%) with common bean (**Supplementary Material**).

To perform the association analysis, we re-filtered our SNP dataset to reduce missing values further (e.g., < 15%) and MAF adjusted 10%. Data imputation was performed to deal with remaining missing data (Frichot et al., 2013; de Villemereuil et al., 2014; Lanes et al., 2018). Gibbs sampler algorithm was run for 10,000 cycles after 5,000 burn-in period in 10 replications. Adjusted p-values distribution and genomic inflation factor (λ = 1,02) were inspected (**Supplementary Material**). False discoveries were controlled using the Benjamin-Hochberg algorithm using *q* = 0.05. To account for unmet model assumptions (e.g., isolation-by-distance or hierarchical population structure), we only considered putative outliers found by both LFMM and *pcadapt* methods (Luu et al., 2017). The *pcadapt* is yet another method implemented in R to detect candidates markers associated with biological adaptation by implementing Principal Component Analysis (PCA) (**Supplementary Material**) (Duforet-Frebourg et al., 2016; Luu et al., 2017).

## Results

### SNP Discovery and Data Processing

The GBS library generated a total of 226,947,346 reads that were retained after multiplexing and quality checking, the sequencing was successful, considering the high number of reads obtained. After demultiplexing and quality control, a total of 673,844 loci were genotyped giving an effective mean coverage per-sample of 12.4× (SD = 8.7). Minimum and maximum coverage per loci were 3.6× and 61.6×, respectively, and the average number of sites per locus of 90.7. After filtering, 28,188 loci were kept composed of 2,597,430 sites from which 61,876 variant sites remained.

### Genomic Diversity

We have found a great variation in *E. heros* nucleotide diversity across the different regions in Brazil. The lowest nucleotide diversity value was found from the northern location PITE (π = 0.0017), and the highest diversity values (π = 0.0062) were found from two central locations MTCA (π = 0.0062) and GOLB (π = 0.0062) (**Table 2**). Observed heterozygosity revealed the same pattern observed of the nucleotide diversity. The inbreeding coefficient (F_IS_) ranged from 0.0009 to 0.0052, showing the populations are largely in equilibrium or outbred rather than inbred.

**Table 2:** Genetic diversity statistics of *Euschistus heros* population from 13 Brazilian locations estimated from RADseq data (193 individuals and 61,876 variant sites included) for all nucleotide positions; H_0_ observed heterozygosity, H_E_ expected heterozygosity, and nucleotide diversity (π) (mean ± SE).

POP	H_O_	H_E_	F_IS_	π
SCAL	0.0025	0.0026	0.0010	0.0028 ± 0.0002
PRPG	0.0044	0.0058	0.0045	0.0056 ± 0.0003
SPAN	0.0044	0.0057	0.0048	0.0059 ± 0.0003
SPPI	0.0044	0.0058	0.0052	0.0059 ± 0.0003
MGPO	0.0044	0.0056	0.0041	0.0059 ± 0.0003
MGUB	0.0043	0.0056	0.0045	0.0059 ± 0.0003
GORV	0.0035	0.0044	0.0030	0.0048 ± 0.0004
GOLB	0.0048	0.0059	0.0039	0.0062 ± 0.0003
MTCA	0.0046	0.0058	0.0042	0.0062 ± 0.0003
MTSO	0.0043	0.0054	0.0037	0.0058 ± 0.0003
MTSI	0.0044	0.0053	0.0034	0.0058 ± 0.0003
TOPA	0.0036	0.0044	0.0031	0.0046 ± 0.0003
PITE	0.0015	0.0016	0.0009	0.0017 ± 0.0001

### Genomic Differentiation and Genetic Structure

**Figure 2:**
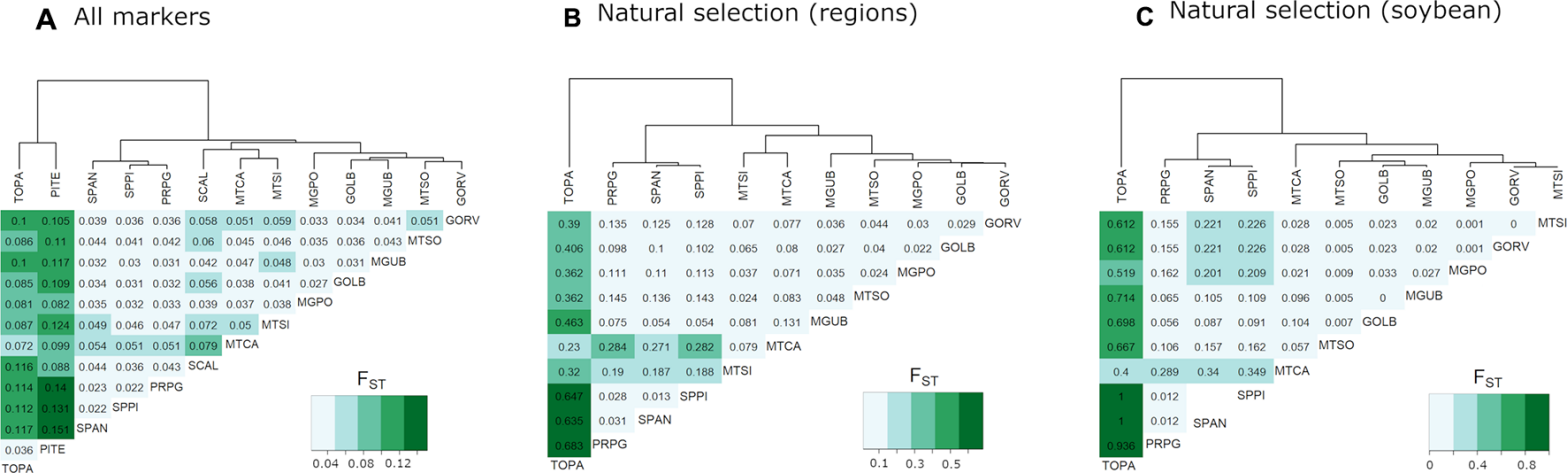
Dendrogram and heatmap based on fixation index values (F_ST_) comparing *E. heros* sampling locations. **(A)** F_ST_ was calculated using the whole set of markers (61,876 SNP) shows separation between northern and southern populations. **(B)** F_ST_ was calculated using candidates putatively under directional selection (30 outliers SNPs) found simultaneously by in Lositan and Bayescan. **(C)** F_ST_ was calculated using a single candidate (27994_38) found associated with the soybean and bean crops gradients detected by all methods tested (Bayescan, Lositan, *pcadapt*, LFMM). Darker color represents a greater degree of differentiation.

The Brazilian populations had a substantial degree of differentiation with F_ST_ = 0.22 (p = 0.000) when comparing northern versus southern locations (**Figure 2A**). Pairwise F_ST_ estimates showed significantly larger values of F_ST_ between northern location (TOPA and PITE) and southern locations (SPPI, SPAN, and PRPG) (**Figure 2A**).

**Figure 3:**
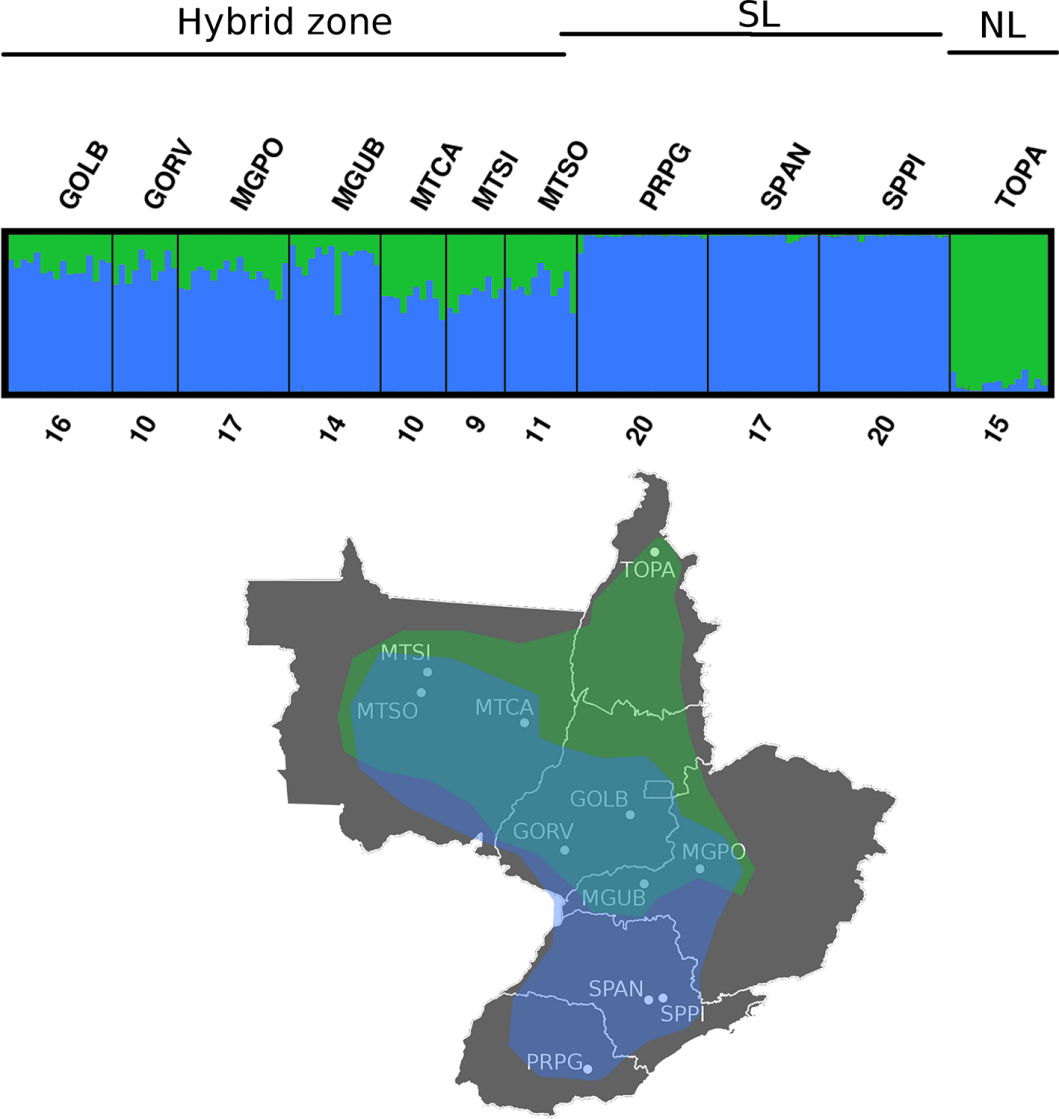
STRUCTURE plot of *E. heros* across the sampled states in Brazil based on 1,565 neutral markers. Vertical bars represent individuals whose genotype have been portioned into 2 clusters. Below is the maps showing the distribution of two putative populations (SL = southern locations and NL = northern locations) and the hybrid zone. Samples from PITE and SCAL were removed due to a low number of shared markers.

**Figure 4:**
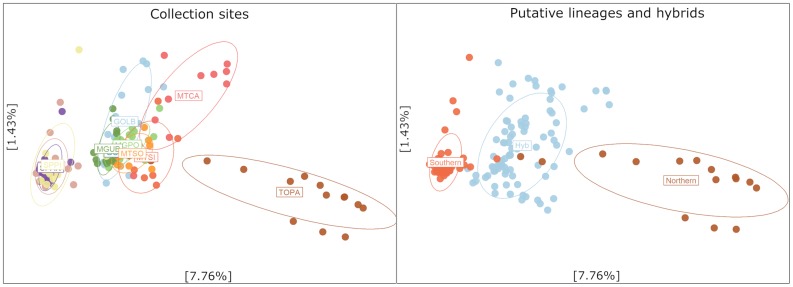
Principal Component Analysis (PCA) based on 20,047 makers showing the separation between northern and southern location and a putative hybrid population.

According to STRUCTURE analysis, the estimated K = 2 was the optimal model of population clustering (**Figure 3**). The northern (NL) and southern (SL) putative lineages can be inferred from the clustering pattern, and the structure model also indicates a fusion zone between lineages at the central region of Brazil with substantial levels of admixture. The hybridization zone in the Cerrado was further confirmed by PCA analysis (**Figure 4**) that showed three clusters, two clusters composed of the purebred from northern and southern populations and a third group composed by hybrids between the two populations present in the Cerrado (**Figure 4**).

**Figure 5:**
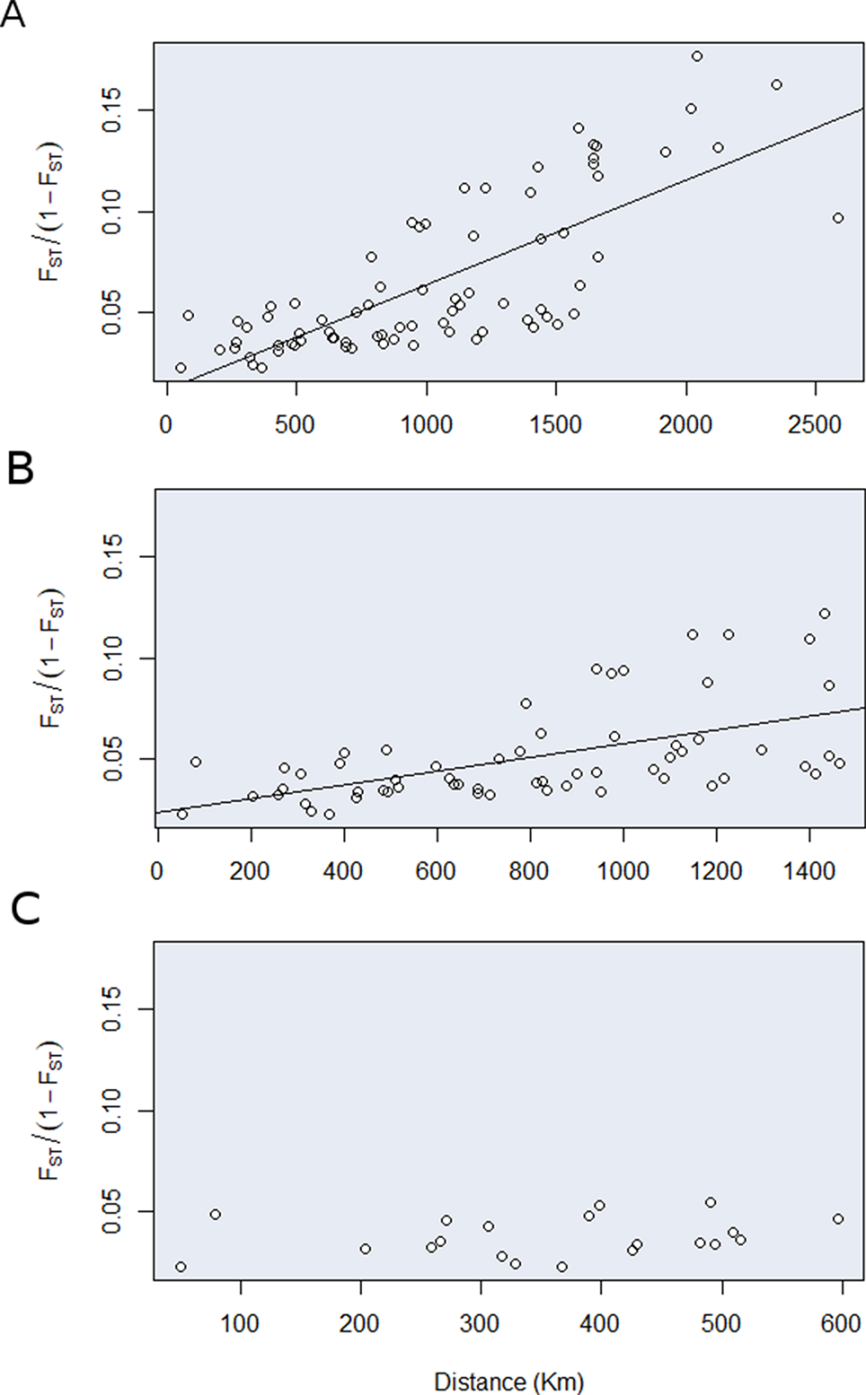
Relationship between genetic and geographic distances of E. heros population based upon the correlation between genetic distance (FST/1-FST) and the geographic distance (km) in three geographic scales: **(A)** 2500, **(B)** 1500, and **(C)** 500 km.

We have also found a significant relationship between genetic and geographical distance (*r* = 0.76, *p* = 0.0001) (**Figure 5**). However, to better separate the effects of isolation by distance and vicariance to gene flow, we tested the association between genetic and geographic distances in three different scales (i.e., 2,600, 1,500, and 500 km). The association between genetic distance and geographic distance became less important as we move to a finer geographic scale. When our data contained northern, southern, and central locations the association was stronger (ß = 5.19 × 10^-5^, *p* = 0.000) compared to the scenario where just the southern and central location (ß = 3.4 × 10^-5^, *p* = 3 × 10^-6^) were included, or when just the central locations where taken into account (ß = 1.7 × 10^-5^, *p* = 0.3). These results indicate that isolation by distance here is likely an artefact due to genetic structure and the variation present in gene pools can be better described as discrete rather than continue across the range of geographic distribution.

### Adaptive Studies

Results from Lositan using the F_ST_ approach to detect outlier loci considering a set of 1,565 SNPs markers identified 161 loci putatively under positive selection. Bayescan detected 73 outliers under positive selection, from which nine can be considered ‘strongly’ under selection and 43 ‘decisively’ under selection. A total of 30 markers fully agree with the results of Lositan. From the 30 unique RAD-tag candidate loci under positive selection detected by both methods, only two had correspondent sequences in the NCBI database (**Table 3**). After generating ST based heatmap, it is possible to infer that the natural selection process seems to be further differentiating the northern location (TOPA), and also increasingly acting on hybrid populations in the Brazilian cerrado such as MTCA and MTSI (**Figure 2B**).

**Table 3:** Outlier loci under positive selection. Listed the radloci ID, the putative gene in the database, identity (%) in relation to the reference sequence, the expected value (e-value) that is the number of hits expected to see by chance for a given sequence size, and Gene Ontology (GO) biological process description.

Locus	Putative gene ID	Identity	e-value	Biological process
4983	Lachesin-like isoform X2*	96	5.6 e^-8^	Neurogenesis
21170	Odorant receptor 4-like**	96	5.6 e^-8^	Olfactory receptor activity
4983	Gamma-aminobutyric acid type B receptor subunit 2*	91	1.66 e^-10^	Component of a heterodimeric G-protein coupled receptor GABA-B
10608	H(+)/cl(-) exchanger transporter 5 isoform x1**	97	2.76 e^-10^	voltage-gated chloride channel activity

*Found only by Lositan.

**Found by Lositan and Bayescan.

#### Association Between Loci and Host Plant Composition in the Landscape

**Figure 6:**
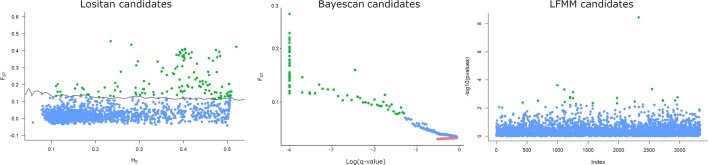
Detection of outlier SNPs under positive selection using FST approach implemented in Lositan, Bayescan, and LFMM. Candidates under positive selection can be identified by the color (green), neutral (Blue), and balancing selection (red). Lositan and Bayescan searched significant outliers contrasting sampled location while FLMM performed a genome-wide test for local adaptation using the soybean crop gradient in the Brazil agriculture landscape.

Using 2 as the latent factor (K = 2) and controlling the false discovery rate (FDR) at q = 0.05, we have not found any SNP associated with the cotton crop gradient (PC2) in sampled areas. However, a total of 18 candidate SNPs were detected associated with soybean (PC1) and 7 associated with the common bean gradients (PC3) (**Figure 6**, **Supplementary Material**). Out of 18 candidate loci with significant correlation with soybean, 5 were also detected by *pcadapt*, and 1 candidate (27994_38) was also detected by all other methods tested (i.e., Lositan, Bayescan, pcadapt, and LFMM) (**Figure 6**). Regarding common bean, out of 7 candidate loci with significant correlation with the common bean gradient, 2 coincided with soybean markers between which one (27994_38) was also detected by all other methods. Blastx results did not return any identification for candidate loci. In summary, our results indicate that all five outlier SNP detection methods agreed that at least one SNP candidate is involved in the process of natural selection associated with soybean and common bean gradients in the Brazilian landscapes.

By calculating the F_ST_ relations between populations using this single markers (27994_38), we were able to see that the separation of the northern area TOPA (land cover: ~76% soybean and ~1% common bean) from the other areas indicates that this marker is, at least in part, associated with local adaptation. Moreover, a genetic substructure involving MTCA (~76% soybean and ~0.4% beans) and the two SP locations where soybean is not intensively cultivated (< 2% soybean and <0.5% common bean) show that this marker is responding more strongly to soybean than the common bean gradient (**Figure 2C**).

## Discussion

### Population Differentiation and Hybrid Zone

Our finding supports the evidence that *E. heros* represents a single species comprised of two well-differentiated populations experiencing ongoing admixture and local adaptation. The two populations found here have direct correspondence and confirm the presence of two deeply divergent lineages co-occurring in South America reported in a previous study using mitochondrial markers (Soares et al., 2018). The two populations are geographically separated in which one lineage occupies the northernmost part of the country (e.g., TOPA and PITE) and the second resides in the southern parts (e.g., SCAL, PRPG, and SPAN). The central region (e.g., GORV, GOLB, MTCA, and MTSO), located at the Cerrado, serves as an admixture zone where the hybrid from both lineages can be found. Diversity was similarly distributed throughout the geographic range with a slight tendency of high values at the overlap and hybridization zone, which could indicate a richer gene pool where the most intensive agriculture areas are now located (Spehar, 1995).

Hybrid zones are narrow geographic regions where genetically distinct population or species encounter each other and offspring with mixed ancestry can be found (Barton and Hewitt, 1985). Hybrid zones are relatively common in ecotones and can encompass a wide range of phenotypic attributes (Barton and Hewitt, 1985; Barton and Bengtsson, 1986). Several mechanisms have been proposed to explain the relative stability of such areas in the aftermath of a recent secondary contact (Mathis et al., 2014) such as the natural selection process favoring hybrids (Moore, 1977), the selection against hybrids (Latour et al., 2014), or the strong selection favoring different alleles at the extremes of the range of geographic distribution resulting in maladaptive individuals at the contact zone (Haldane, 1948; Corrêa et al., 2019). In the case of *E. heros*, frequent cases of outbreaks have been reported in the Cerrado region where the secondary contact is occurring (Soares et al., 2018). That could be an indication that hybrids between the two populations might be associated with those outbreaks and have similar or even greater fitness compared to the pure ancestral populations, even though that hypothesis have not been tested yet (Corrêa et al., 2019). The secondary contact probably happened prior to the soybean expansion in the Cerrado during the 1970s (Spehar, 1995; Soares et al., 2018), but large crop fields are favoring the mixing of the two populations by sustaining larger densities of insects in areas intermediary to the two lineages distribution. It is possible that the limited dispersal behavior of this species (suggested by the well-preserved degree of genetic structure) and the natural selection at the extremes of the range of geographic distribution might have prevented the full admixture of the two lineages; however, no dispersal or between-populations fitness comparison estimates are available for this species preventing us from making further inferences. Even though this study encourages new questions, we can, at least for now, refute the hypothesis of human-aid movement fully mixing *E. heros* populations in Brazil and hypothesize the adaptive character of hybridization in this species (Husch et al., 2018).

### Strong Structure and Limited Movement

The distinction between historical events (e.g., climatic changes, population fragmentation, range expansion, or bottlenecks/founder events) and recurrent events (e.g., gene flow, genetic drift and system of mating) have been a point of debate in population genetics over the years and more recently in the field of molecular ecology (Sanmartín, 2003; Meirmans, 2012). The common methods for testing the association between genetic and geographic distances can lead to biased results and ultimately incorrect conclusion regarding the importance of isolation by distance. Both isolation by distance or vicariance to gene flow can result in a significant pattern of association between genetic and geographic distance commonly tested with mantel test (Manel and Holderegger, 2013). We performed successive tests gradually excluding the most geographically distant populations and verified that the strength of the association first decreased, when the northern populations were excluded, then disappeared after we excluded the southern populations. Thus, the results demonstrate that the correlation between genetic and geographic distance is largely due to the presence of structure. At the finer scale (i.e., < 500 km), populations seem to be mixing freely, but we would expect an extremely low number of immigrants to be exchanged at the larger scale (i.e., between the southern and northernmost locations). Those results suggest that even though a great degree of admixture can be found in the Cerrado, newly emerged adaptations would only slowly spread to distant places.

### Adaptive Changes

We have detected a number of DNA markers under strong selection that might be an indication of the transformation that the *E. heros* populations are undergoing in Brazil. Yet, to avoid pitfalls, we must discuss the two major modes of selection, balancing and positive selection, taking in account temporal, spatial, and the biological context (Hohenlohe et al., 2010). Positive selection is expected to reduce genetic diversity, shorten the coalescence time, and increase the frequency of one allele. On the other hand, balance selection is expected to maintain polymorphism, causing higher levels of nucleotide diversity and extended coalescence time (Charlesworth et al., 1997). In the case of *E. heros* populations, overall high levels of genetic diversity have been found, but compelling evidence of hard sweeps selection acting on specific parts of the genome could be linked to the rapid evolution of adaptations in response to management practices. Mutation in GABA receptors (GABA-R) is a possible mechanism that can lead to insecticide resistance in insects (Ffrench-Constant et al., 1993), and insecticides molecules such as avermectins and phenylpyrazole derivate could potentially be involved in the resistance evolution of the GABA-R (Deng and Casida, 1992; Casida and Durkin, 2015). However, those insecticides do not target hemipterans in specific; therefore, it is unclear to us how and what processes are causing the evolution of chloride channels and GABA receptors in *E. heros*. Historically, cyclodienes (e.g., α-endosulfan) were largely used to control stinkbugs, but the molecular mechanism of action is still not well understood to this day. Moreover, this insecticide group has restricted usage in Brazil (Sebastian and Raghavan, 2017).

Interestingly, an odorant receptor was also listed as a candidate for positive selection in *E. heros* populations, and that could be a preliminary indication of a host selection process evolution (An et al., 2016). However, functional studies are needed to confirm this hypothesis. Based on F_ST_ analysis of markers detected by both Bayescan and Lositan a great differentiation between the northern location POTA and all other locations could still be noticed indicating that not only genetic drift but the natural selection helped shape the features of modern populations. A variety of adaptive differences are expected, giving the differences in environmental conditions and landscape composition found in POTA and PRPG, for instance.

### Host Expansion and Soybean/Cotton Adaptations

*Euschistus heros* has been ubiquitous in soybean fields for many years, but just recently have been increasingly reported in cotton fields. In our association analysis, we have not found a significant association between allele frequencies and the gradient of cotton crops in the agriculture landscape of Brazil. On the other hand, we found 14 candidate SNPs responding to the soybean and 7 to beans gradients. Although still not clear by which mechanism, a single marker (27994_38), detected by all tested methods, seems to be associated with both soybean and common bean crops. Locations where soybean crops are the most abundant (POTA and MTCA) reaching up to 76% of the covered area, and in places where it is almost completely absent (SPAN and SPPI), helped form a pattern of substructure based on this marker. However, we still cannot completely discard possible loci association with cotton crops as our sampled were not broad enough to cover areas with high cotton prevalence (e.g., > 40%). Moreover, in our samples, cotton crops were intermingled with soybean crops, which can create unaccounted interactions between *E. heros* genotypes and the environmental gradients.

Those results suggest host range expansion rather than host shift is likely the process by which *E. heros* is becoming more prevalent in cotton crops. Host plant shift can be defined as the process by which the organism abandoned one or more host species in favor or a new host (Tabashnik, 1983), which is distinct from the host range expansion that occurs when the herbivores increase the number of potential hosts (Tabashnik, 1983). We can infer that insects that feed on cotton belong to the same gene pool of insects feeding on soybean and common bean with few, but perhaps key differences. The exploitation of a new host species can be explained by several mechanisms, but an often-used explanation is the improvement of the detoxification capability (Mullin and Croft, 1983; Matzkin, 2008). In some cases, host shift or host expansion can occur simply by the recombination of existing adaptations, which is the most like the case for *E. heros* (Lefeuvre and Moriones, 2015). The recombination of genes from northern populations that are usually associated with common bean crops and native plants with southern populations that are strongly linked with soybean crop can be proposed as a hypothesis to explain *E. heros* host expansion in the Cerrado. To test this hypothesis, one has to investigate behavioral and functional differences using individuals collected in different crops in a more systematized design to reduce confounding factors (Raszick et al., 2019).

### The Implication for Pest Management

Both the domain of the two lineages and the mixing zone should be taken into account during the decision-making process regarding the adoption of new management plans and technologies aiming soybean and cotton protection. For instance, the evaluation of susceptibility of new plant varieties should ideally be tested in both extreme environments (northern and southern locations). Unpredictable results might be expected in the central region where the two populations are exchanging adaptions. Resistance evolution, host adaptation, and changes in the dormancy behavior are expected to evolve first in the Cerrado. At the same time, the spreading of resistance or other adaptations to other areas is expected to happen slowly given the limited gene flow between the two areas.

In conclusion, one of the direct benefits attributed to the adoption of *Bt* plants in Brazil was the sensitive reduction in the frequency of insecticide applications targeting lepidopterans. However, an inconvenient side effect of such adoption is the now frequent hemipterans outbreak that has become an undesirable trend in soybean field throughout the Cerrado. Here, we provided shreds of evidence that the admixture of the two *E. heros* lineages and possible the evolution of adaptations associated with host recognition and increase in survival and reproduction might why *E. heros* is so dominant in the soybean ecosystems. The results presented here provide a valuable foundation for future studies to elucidate the mechanisms by which host range and resistance evolutions are affecting natural populations aiding the development of effective tools for pest control.

## Author Contributions

MZ conceived and designed the study, generated the molecular data, conducted the data analysis, and drafted the manuscript. EC conducted the data analysis, and drafted the manuscript. XW conducted the data analysis. LML conducted the data analysis on lab. PB conducted the data analysis and the manuscript review. SM conducted the data analysis on lab. JV conducted the data analysis. CO conducted the field sampling and the manuscript review. JP conducted the field sampling and the manuscript review. SC conceived and designed the study, and manuscript review.

## Conflict of Interest

The authors declare that the research was conducted in the absence of any commercial or financial relationships that could be construed as a potential conflict of interest.
