# Supplementary material for: Population Genomics of the Neotropical Brown Stink Bug, Euschistus heros: The Most Important Emerging Insect Pest to Soybean in Brazil
Source: Front Genet. 2019 Oct 31;10:1035. doi: 10.3389/fgene.2019.01035 (PMC6844245; doi:10.3389/fgene.2019.01035)
Supplement: Supplementary file 1 [file DataSheet_1.docx]

**FRONTIER in GENETICS**

**Supplementary material**

**Population Genomics of the Neotropical brown stink bug, *Euschistus heros*: The most important emerging insect pest to soybean in Brazil**

**Maria I. Zucchi, Erick M. G. Cordeiro,** Xing Wu, Letícia Marise Lamana, Patrick J. Brown, Shilpa Manjunatha, Celso Omoto**,** J. Baldin Pinheiro, Steven J. Clough


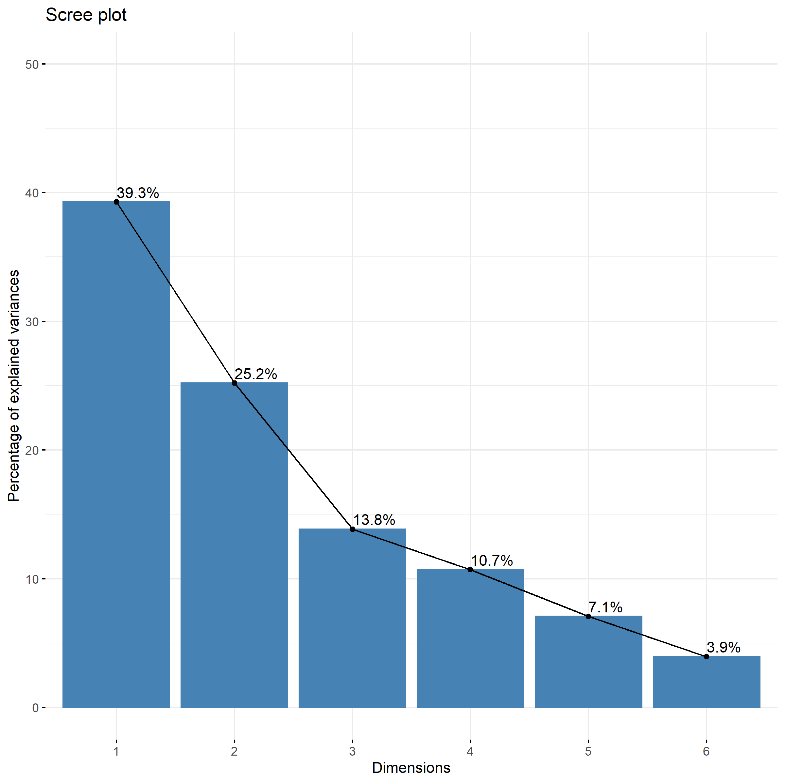


**Figure 1.** Percentage of the explained variance by each principal component axis.

**
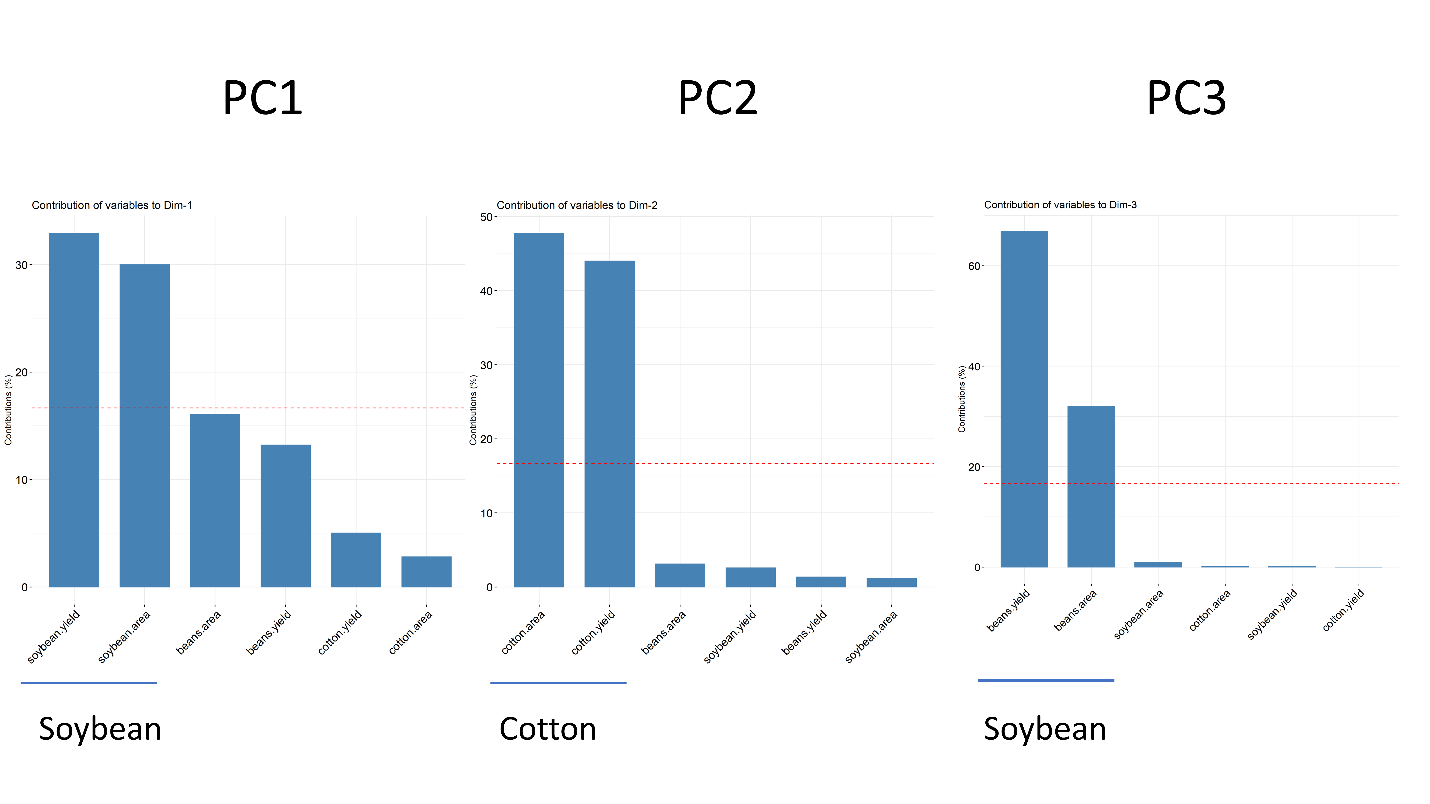
**

**Figure 2.** Variable contribution to first, second and third PC axis.

**
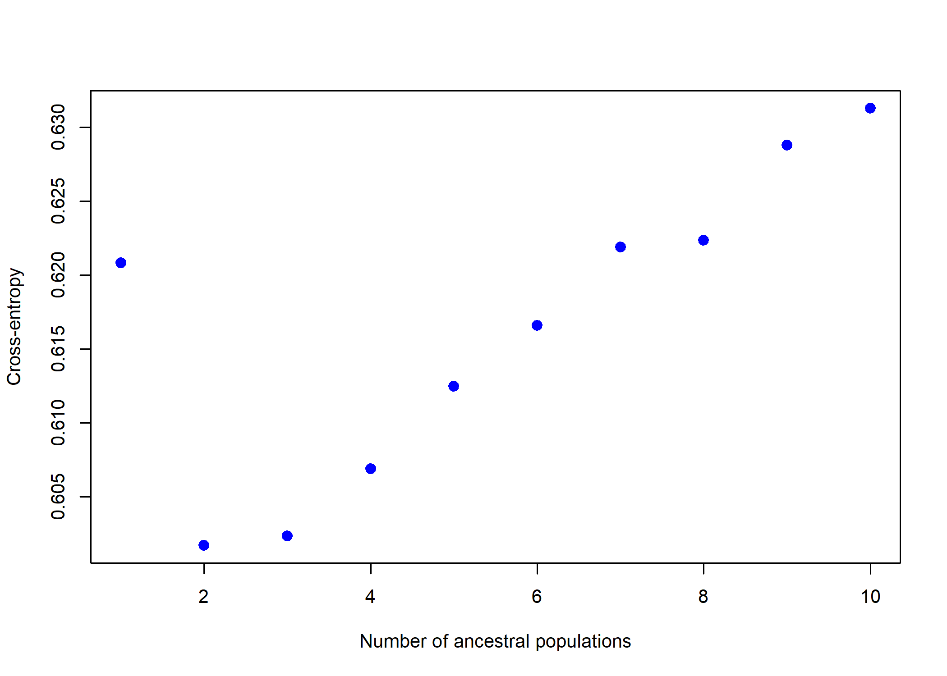
**

**Figure 3.** Values of cross-entropy criterion for each K value using *snmf*. The value for which K is observed at its minimum value is 2.

**
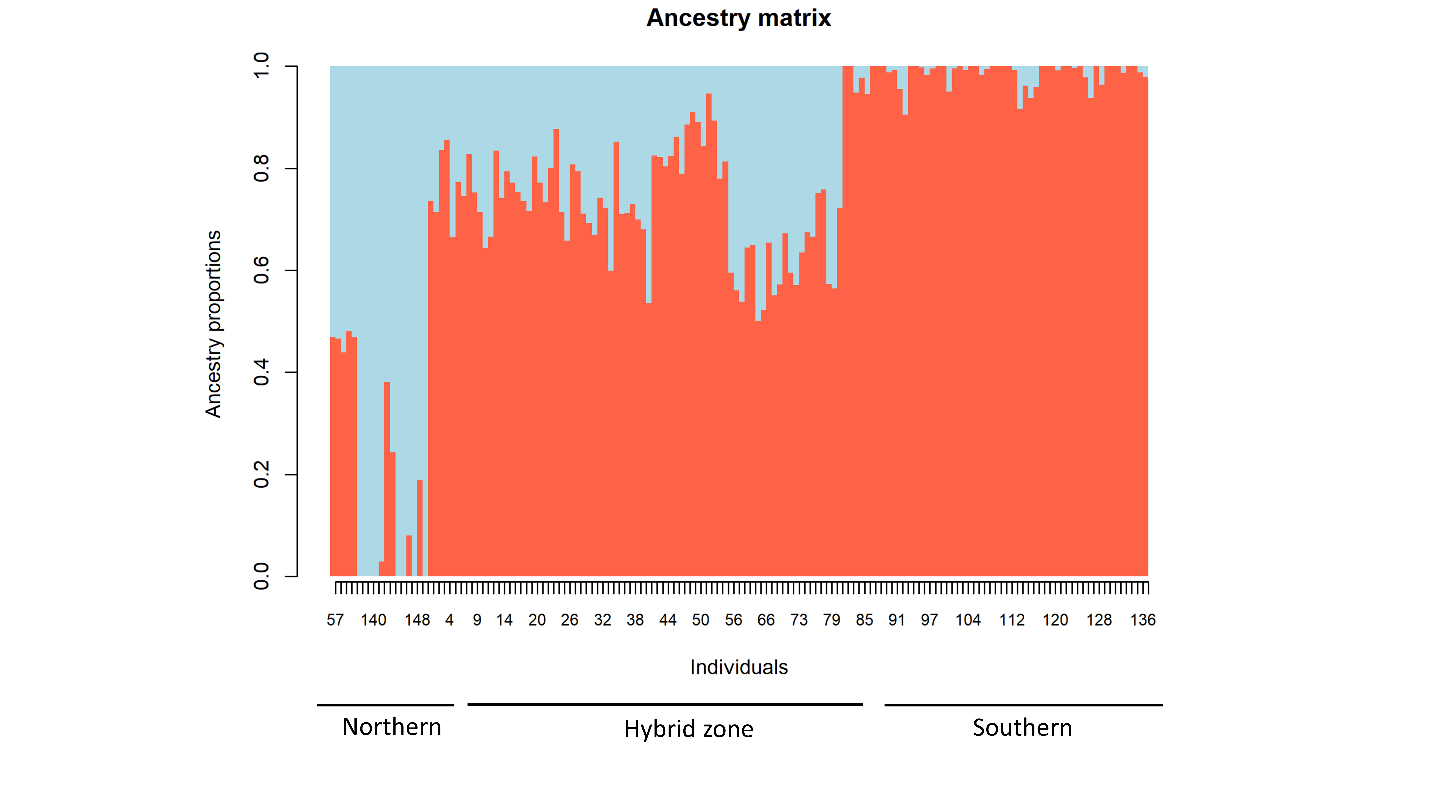
**

**Figure 4.** Barplot of ancestry coefficients based on the number of factors 2 (K=2). Ancestry pattern of sampled individuals confirms STRUCTURE analysis.

**
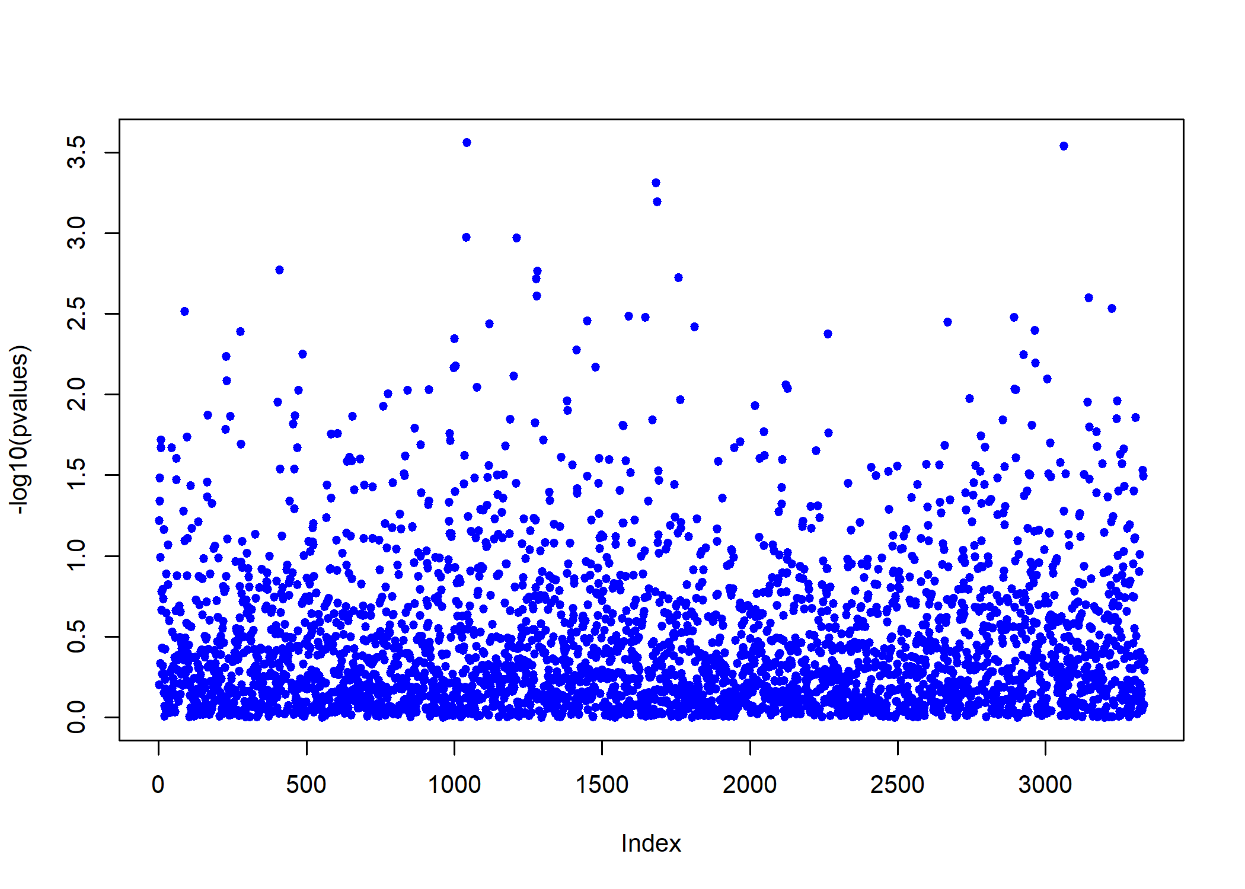
**

**Figure 5.** Manhattan plot showing SNP associations between genetic markers and environmental values for bean crops retrieved from PC3. A total of 7 markers were significantly associated with bean crops.

**
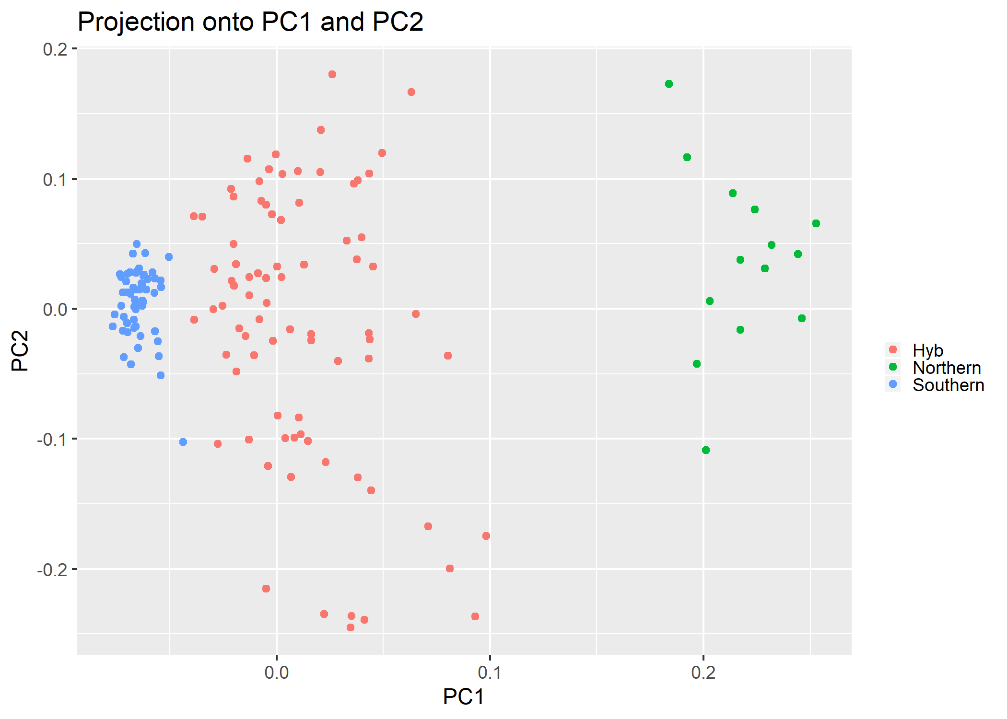
**

**Figure 6.** Principal component analysis (PCA) between PC1 and PC2 showing the separation between northern, southern, and hybrids.

**
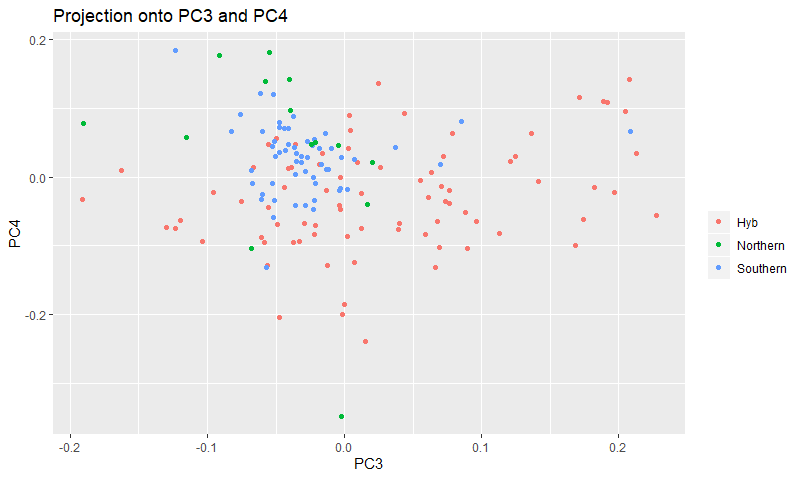
**

**Figure 7.** Principal component analysis (PCA) between PC3 and PC3 showing that informative information has been captured by PC1 and PC2.

**
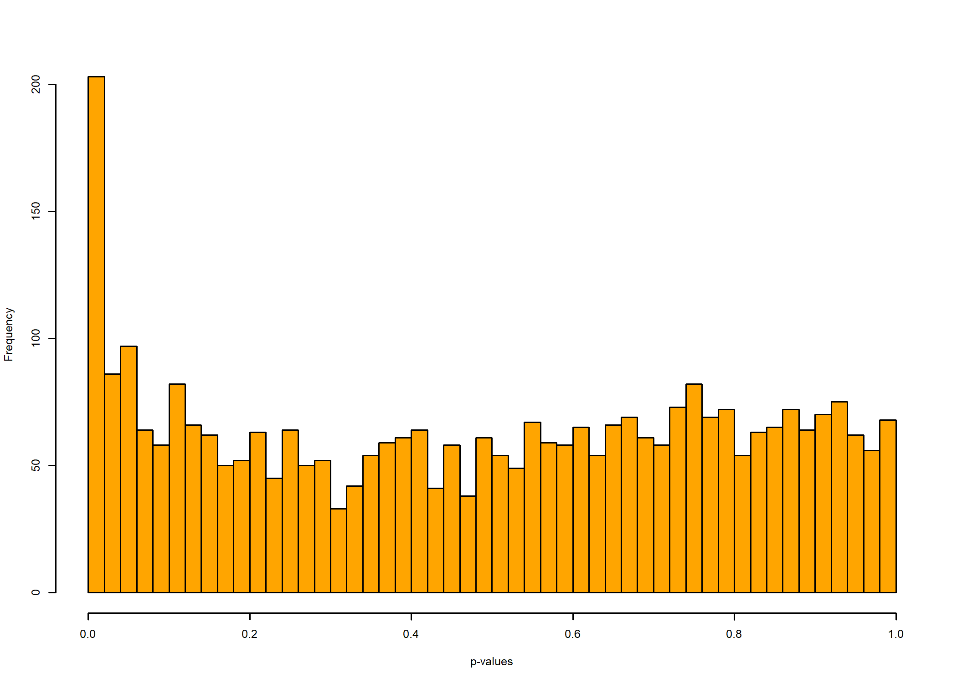
**

**Figure 8.** Histogram of p-values confirming that most values follow a uniform distribution.

**
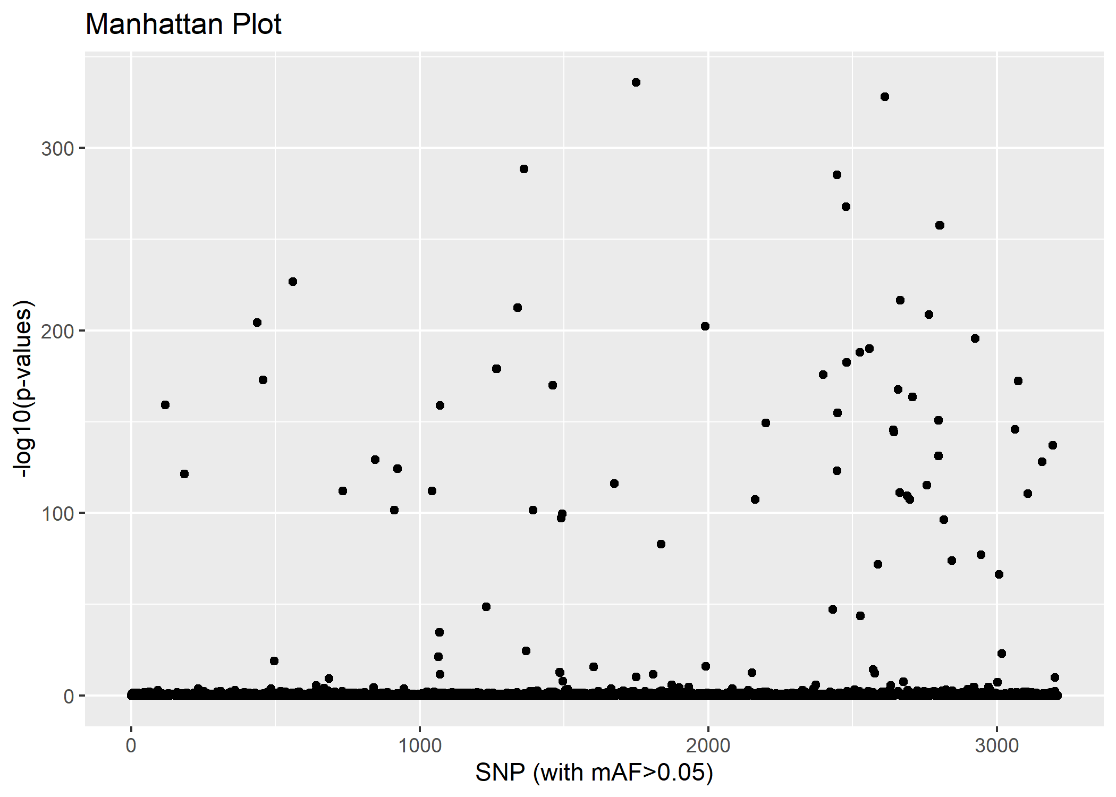
**

**Figure 9.** Manhattan plot showing *pcadapt* outlier candidates.
